# Supplementary material for: Computational design of dynamic receptor—peptide signaling complexes applied to chemotaxis
Source: Nat Commun. 2023 May 19;14:2875. doi: 10.1038/s41467-023-38491-9 (PMC10198977; doi:10.1038/s41467-023-38491-9)
Supplement: Supplementary file 3 — Reporting Summary [file 41467_2023_38491_MOESM3_ESM.pdf]

nature portfolio

Corresponding author(s): Patrick Barth

Last updated by author(s): Apr 19, 2023

Reporting Summary

Nature Portfolio wishes to improve the reproducibility of the work that we publish. This form provides structure for consistency and transparency in reporting. For further information on Nature Portfolio policies, see our Editorial Policies and the Editorial Policy Checklist.

Statistics

For all statistical analyses, confirm that the following items are present in the figure legend, table legend, main text, or Methods section.

n/a	Confirmed	
☐	☒	The exact sample size (*n*) for each experimental group/condition, given as a discrete number and unit of measurement
☐	☒	A statement on whether measurements were taken from distinct samples or whether the same sample was measured repeatedly
☐	☒	The statistical test(s) used AND whether they are one- or two-sided<br>*Only common tests should be described solely by name; describe more complex techniques in the Methods section.*
☒	☐	A description of all covariates tested
☐	☒	A description of any assumptions or corrections, such as tests of normality and adjustment for multiple comparisons
☐	☒	A full description of the statistical parameters including central tendency (e.g. means) or other basic estimates (e.g. regression coefficient) AND variation (e.g. standard deviation) or associated estimates of uncertainty (e.g. confidence intervals)
☐	☒	For null hypothesis testing, the test statistic (e.g. *F*, *t*, *r*) with confidence intervals, effect sizes, degrees of freedom and *P* value noted<br>*Give P values as exact values whenever suitable.*
☒	☐	For Bayesian analysis, information on the choice of priors and Markov chain Monte Carlo settings
☒	☐	For hierarchical and complex designs, identification of the appropriate level for tests and full reporting of outcomes
☒	☐	Estimates of effect sizes (e.g. Cohen's *d*, Pearson's *r*), indicating how they were calculated

Our web collection on statistics for biologists contains articles on many of the points above.

Software and code

Policy information about availability of computer code

Data collection	BRET was measured on a Mithras2 LB 943. Luminescence and fluorescence were measured on a Molecular Devices FlexStation3. Computational data were generated using protein modeling, docking, and design applications (RosettaMembrane, FlexPepDock) available in the v3.13 of the Rosetta software package (https://www.rosettacommons.org/software) and scripts for peptide-receptor modeling and design available on GitHub (https://github.com/barthlab/CAPSens_design). Molecular dynamics simulations were prepared with the CHARMM-GUI web server v3.8 (https://www.charmm-gui.org/?doc=input) and performed using the GROMACS software package v2020.4 (https://www.gromacs.org/).
Data analysis	In vitro assays were analyzed using GraphPad Prism v9. Computational data were analyzed using standard clustering programs available in v3.13 of the Rosetta software package (https://www.rosettacommons.org/software). Molecular dynamics trajectories were analyzed using AlloDy (https://github.com/barth-lab/AlloDy). Receptor cavity geometry was analyzed using pyKVFinder v0.4.0 (https://github.com/LBC-LNBio/pyKVFinder).

For manuscripts utilizing custom algorithms or software that are central to the research but not yet described in published literature, software must be made available to editors and reviewers. We strongly encourage code deposition in a community repository (e.g. GitHub). See the Nature Portfolio guidelines for submitting code & software for further information.

Data

Policy information about availability of data

All manuscripts must include a data availability statement. This statement should provide the following information, where applicable:

- Accession codes, unique identifiers, or web links for publicly available datasets
- A description of any restrictions on data availability
- For clinical datasets or third party data, please ensure that the statement adheres to our policy

The authors declare that all data supporting the findings in this study are either presented within the article and its Supplementary Information files or available from the corresponding author on request. Source data are provided with this paper. The following PDB entries were used for modeling: 4RWS, 4XT1, 6LFO, 4UAI, and 7F1R.

Human research participants

Policy information about studies involving human research participants and Sex and Gender in Research.

Reporting on sex and gender	No human research participants were used in this study.
Population characteristics	No human research participants were used in this study.
Recruitment	No human research participants were used in this study.
Ethics oversight	No human research participants were used in this study.

Note that full information on the approval of the study protocol must also be provided in the manuscript.

Field-specific reporting

Please select the one below that is the best fit for your research. If you are not sure, read the appropriate sections before making your selection.

☒ Life sciences ☐ Behavioural & social sciences ☐ Ecological, evolutionary & environmental sciences

For a reference copy of the document with all sections, see nature.com/documents/nr-reporting-summary-flat.pdf

Life sciences study design

All studies must disclose on these points even when the disclosure is negative.

Sample size	For receptor dose responses, at least 2 technical replicates were performed to achieve a significant response compared to the mock control. For measurement of EC50 and maximum activity, experiments were repeated at least 3 times to have confidence in reproducible parameters of dose responses. For migration assays, 3 technical replicates were performed for at least 3 independent groups of donor-derived T cells in order to measure statistical significance to the WT control.
Data exclusions	No data were excluded from the analysis.
Replication	All attempts at replication were successful. Dose response data was replicated at least 2 times. Migration assays were replicated in two independent rounds of experimentation with 3 groups of donor-derived T cells each.
Randomization	All buffy coat samples were from de-identified healthy donors and considered to be from the same group and thus not randomized. Transduction efficiencies were compared to non-transduced samples, and migration responses were compared to WT-transduced samples.
Blinding	All buffy coat samples were de-identified and were all from healthy donors, so blinding of group allocation was not relevant.

Reporting for specific materials, systems and methods

We require information from authors about some types of materials, experimental systems and methods used in many studies. Here, indicate whether each material, system or method listed is relevant to your study. If you are not sure if a list item applies to your research, read the appropriate section before selecting a response.

Materials & experimental systems

n/a	Involved in the study
☐	☒ Antibodies
☐	☒ Eukaryotic cell lines
☒	☐ Palaeontology and archaeology
☒	☐ Animals and other organisms
☒	☐ Clinical data
☒	☐ Dual use research of concern

Methods

n/a	Involved in the study
☒	☐ ChIP-seq
☒	☐ Flow cytometry
☒	☐ MRI-based neuroimaging

Antibodies

Antibodies used	Antibodies used to perform "cell surface ELISA" were against HA (Thermofisher, ref:26183, clone: 2-2.2.14) and HRP-tagged anti-mouse IgG (CST, ref:7076S). Anti-CD3 (Biolegend, ref: 317347, clone: OKT3) and anti-CD28 (Biolegend, ref: 302934, clone: CD28.2) were used to generate activated T cells. PE-conjugated anti-HA (Biolegend, ref: 901518, clone: 16B12) was used to select for transduced T cells.
Validation	All antibodies are well known in the literature for their specificity. As well, a negative control of cells transfected with an empty plasmid was used for background signal. These data are shown in Supplementary Fig. 2.

Eukaryotic cell lines

Policy information about cell lines and Sex and Gender in Research

Cell line source(s)	HEK 293T cells from Ted Wensel at Baylor College of Medicine were used for all BRET, ELISA, and calcium mobilization assays. Peripheral blood mononuclear cells were isolated from buffy coats of de-identified healthy human volunteer blood donors obtained from the Center of Interregional Blood Transfusion SRK Bern (Bern, Switzerland).
Authentication	No cell line authentication.
Mycoplasma contamination	Cells were regularly tested for mycoplasma contamination (qPCR, Eurofins Genomics). Results were conistently negative for mycoplasma.
Commonly misidentified lines (See ICLAC register)	No commonly misidentified lines.
